# DNA Methylation and Transcriptomic Changes in Response to Different Lights and Stresses in *7B-1* Male-Sterile Tomato

**DOI:** 10.1371/journal.pone.0121864

**Published:** 2015-04-07

**Authors:** Vahid Omidvar, Martin Fellner

**Affiliations:** Group of Molecular Physiology, Laboratory of Growth Regulators, Palacky University & Institute of Experimental Botany ASCR, Olomouc, Czech Republic; Henan Agricultural Univerisity, CHINA

## Abstract

We reported earlier that *7B-1* mutant in tomato (*Solanum lycopersicum* L., cv. Rutgers), an ABA overproducer, is defective in blue light (B) signaling leading to B-specific resistance to abiotic and biotic stresses. Using a methylation-sensitive amplified polymorphism (MSAP) assay, a number of genes were identified, which were differentially methylated between *7B-1* and its wild type (WT) seedlings in white (W), blue (B), red (R) lights and dark (D) or in response to exogenous ABA and mannitol-induced stresses. The genomic methylation level was almost similar in different lights between *7B-1* and WT seedlings, while significant differences were observed in response to stresses in D, but not B. Using a cDNA-AFLP assay, several transcripts were identified, which were differentially regulated between *7B-1* and WT by B or D or in response to stresses. Blue light receptors *cryptochrome 1* and *2* (*CRY1* and *CRY2*) and *phototropin 1* and *2* (*PHOT1* and *PHOT2*) were not affected by the *7B-1* mutation at the transcriptional level, instead the mutation had likely affected downstream components of the light signaling pathway. 5-azacytidine (5-azaC) induced DNA hypomethylation, inhibited stem elongation and differentially regulated the expression of a number of genes in *7B-1*. In addition, it was shown that mir167 and mir390 were tightly linked to auxin signaling pathway in 5-azaC-treated *7B-1* seedlings via the regulation of auxin-response factor (*ARF*) transcripts. Our data showed that DNA methylation remodeling is an active epigenetic response to different lights and stresses in *7B-1* and WT, and highlighted the differences in epigenetic and transcriptional regulation of light and stress responses between *7B-1* and WT. Furthermore, it shed lights on the crosstalk between DNA hypomethylation and miRNA regulation of *ARF*s expression. This information could also be used as a benchmark for future studies of male-sterility in other crops.

## Introduction

The *7B-1* mutant in tomato (*Solanum lycopersicum* L., cv. Rutgers) is a genic photoperiod-dependent male-sterile in long days with stamens that are shrunken and produce non-viable microspores [1], while in short days flowers are fertile and produce normal stamens and viable pollens. In *7B-1*, microsporogenesis breaks down prior to the meiosis in microspore mother cells (MMC). A proteomic study showed that large number of proteins with important roles in tapetum and MMC developments were differentially modulated in *7B-1* anthers during the meiosis [2]. Compared to the WT, *7B-1* is less sensitive to light-induced inhibition (i.e., de-etiolation) of hypocotyl growth, has a higher endogenous ABA level, but less GAs, IAA, CKs and is more tolerant to various abiotic stresses, specially under blue light [3–5]. A study by Fellner and Sawhney [4] suggested a defect in blue light perception in *7B-1*, which in turn affected hormonal sensitivity and their endogenous level. Being a photoperiod-dependent male sterile and stress tolerant, the *7B-1* mutant offers an exceptionally attractive germplasm for hybrid tomato breeding [6].

Recently, there have been growing interests toward understanding the epigenetic regulation of plant development and response to environmental cues, such as light and stresses [7,8]. Epigenetics is defined as the heritable alteration of gene expression without changing the basic DNA sequence [9]. These alterations, such as DNA methylation, histone modifications, and small RNA interference can play an individualized role or work in concert to regulate plant responses to environmental cues. DNA methylation is important in regulating gene expression and in silencing transposons and other repetitive sequences and is catalyzed by cytosine methyltransferases, which can occur in three sequence contexts: CG, CHG and CHH (H = A, T, or C). In some cases, methylated cytosine residues in promoter and enhancer regions may directly prevent the binding of transcription factors, but in most cases, the presence of methylated cytosine is thought to attract methylcytosine-binding proteins, which recruit histone deacetylases and chromatin remodeling proteins that in turn compact the chromatin and restrict access of the transcription machinery [10]. The *Arabidopsis* genome has 24% of CG, 6.7% of CHG and 1.7% of CHH sites methylated at the cytosine [11], with transposons and DNA repeats comprising the largest fraction of methylated DNA sequences. Transcriptional gene silencing is usually associated with methylation of the gene promoter regions, while methylation of coding regions does not result in gene silencing, and even occasionally has a positive effect on gene expression [12]. Genome-wide analysis revealed that about one-third of *Arabidopsis* genes contain methylated cytosines in their coding regions [11,12].

An increasing trend of DNA methylation level during plant growth and development has been reported in *Arabidopsis*, tomato, and rice [8,13,14], however aberrant methylation could impede normal development and reduce fertility as demonstrated in *MET1* loss of function of *Arabidopsis* [15]. Li et al. [16] showed that different light qualities resulted in distinct DNA methylation variations in cotton. Expression of a photoperiod-responsive gene in rice was regulated by DNA demethylation induced by short day photoperiod [17]. In a photoperiod-sensitive male-sterile rice, *de novo* methylation of a promoter reduced the expression of a non-coding RNA leading to male sterility under long day conditions [18]. *Stellaria longipes* treated with low red/far-red light ratios showed a lower level of methylation, which was a crucial factor in controlling the stem elongation response [19]. In response to pathogen attack in tomato, DNA methylation was altered largely in the genomic regions involved in defense and stress responses [20]. In response to drought, expression of the stress-inducible *Asr1* gene in tomato was induced concurrently with decrease in the intragenic methylation level [21]. DNA methylation level was significantly decreased in maize upon cold treatment [22,23] and in rice roots in response to salinity [24]. In tobacco, *GPDL* and *Alix1* genes were demethylated and up regulated as a result in response to aluminum stress and tobacco mosaic virus infection, respectively [25,26]. The pea genome was hypermethylated in response to water deficit stress [27]. Methylation level in *Mesembryanthemum crystallinum* genome increased as an adaptive response to high salinity condition [28].

DNA methylation can be detected via bisulfite conversion, methylation-sensitive restriction enzymes, methyl-binding proteins and anti-methylcytosine antibodies. Methylation-sensitive amplified polymorphism (MSAP) technique has been successfully applied to study DNA methylation variations in many plants, including tomato [20,21,28]. The main objective of this study was to investigate the differences in DNA methylation and transcriptional regulation in response to different lights and stresses between *7B-1* and WT, which were associated with the *7B-1* mutation and male-sterility in *7B-1*. DNA methylation localization across the genome was profiled in *7B-1* and WT seedlings in different light conditions and in response to exogenous ABA and mannitol using the MSAP approach. Global changes of the genomic methylation level were also measured in *7B-1* and WT seedlings in the above mentioned conditions using anti-methylcytosine antibodies. Transcriptomic changes in blue light, dark, and in response to ABA, mannitol, and also 5-azaC (DNA demethylating agent) were studied in *7B-1* and WT seedlings using a cDNA-AFLP assay.

## Materials and Methods

### Plant material and stress treatments

Tomato seedlings were grown either on a basal MS medium [29] or in soil. Seedlings were grown under continuous W, B, and R lights or in D in temperature-controlled growth chambers set at 23°C (Microclima 1000E, Snijders Scientific B.V., The Netherlands) equipped with different light sources. W was provided by white cool fluorescent tubes (Philips TLD-36W/54, Phillips, USA). B and R were provided by blue (Philips TLD-36W/18-Blue) and red (Philips TLD-36W/15-Red) fluorescent tubes with a maximum irradiance at 460 and 660 nm, respectively. The total photon fluence rate was calibrated to 10 *μmol m* ^*-2*^ s^*-1*^ by the Department of Biophysics at Palacky University in Olomouc using a portable spectroradiometer (model LI-1800, LI-COR, Lincoln, Nebr.). Stress treatments were carried out as described by Fellner and Sawhney [4] by growing the seedlings on MS mediums containing 10 μM ABA or 140 mM mannitol or 10 μM fluridone (an inhibitor of ABA biosynthesis). 5-Azacytidine treatment was carried out by placing drops of 40% ethanol solution containing 50 uM 5-azaC directly to the shoot apical meristem of 2-month old seedlings grown in long days (16 h light) once a day for 30 days. Control seedlings were treated similarly, but using ethanol drops instead of 5-azaC. Three biological replicates of WT and *7B-1* were included for all the treatments.

### MSAP analysis

Genomic DNA was extracted from the biological replicates using the DNeasy Plant Mini kit (Qiagen), and pooled in equimolar ratio. MSAP analysis was performed as described by Portis et al. [30]. List of adapter sequences and primers used for pre- and selective PCR amplifications is provided in S1 Table. In brief, 200 ng of genomic DNA was digested using 20 U of either *Hpa*II or *Msp*I and 10 U of *Eco*RI (New England Biolabs) in a final volume of 20 μl for 4 h at 37°C. Digested DNAs were then ligated to *Eco*RI and *Hpa*II/*Msp*I adapters and preamplified using primers complementary to the adapter sequence plus a single base extension (Eco+A and H/M+T). Primary PCR products were diluted 10 x and used as templates for selective PCRs using 24 primer combinations. MSAP products were separated on 6% sequencing acrylamide gels and visualized by silver staining.

### Measuring global genomic methylation level

Global genomic methylation level was measured using the MethylFlash Methylated DNA Quantification kit (Epigentek). In brief, DNA was first bound to high affinity strip wells, and then the methylated fraction of DNA was detected using capture/detection antibodies and subsequently quantified in an ELISA-based assay by reading the absorbance at 450 nm. The amount of methylated DNA is proportional to the absorbance reading. Level of 5-methylcytosine was quantified as the percentage of methylated cytosine in total genomic DNA using the formulas described in the kit manual.

### cDNA-AFLP analysis

Total RNA was extracted from the biological replicates using the RNeasy Plant Mini kit (Qiagen), and pooled in equimolar ratio. First-strand cDNAs were synthesized using the PrimeScript First Strand cDNA Synthesis kit (TAKARA). Second strand cDNAs were synthesized using the NEBNext mRNA Second Strand Synthesis kit (New England Biolabs), and used for cDNA-AFLP analysis following the protocol described by Bachem et al. [31]. List of adapter sequences and primers used for pre- and selective PCR amplifications is provided in S2 Table. In brief, 100 ng of double stranded cDNAs were digested with *Eco*RI and *Mse*I. Digested products were then ligated to *Eco*RI and *Mse*I adapters. Pre-amplification was carried out using *Eco*RI and *Mse*I adapter-specific primers. Pre-amplified reactions were diluted 10 x and used as templates in selective PCRs using 24 primer combinations. AFLP products were separated on 6% sequencing acrylamide gels and visualized by silver staining.

### Cloning and characterization of differentially amplified fragments

Differentially amplified fragments were isolated from the gel following the procedure described by Wang et al. [24] and reamplified using same sets of primers used for the selective PCR. Amplified fragment were subsequently cloned into the pGEM-T Easy Vector (Promega) and subjected to sequence analysis. Sequences were annotated using blast search against the SOL Genomics Network (SGN; http://solgenomics.net/) and NCBI databases and gene ontologies were assigned from SGN, EBI (http://www.ebi.ac.uk/interpro/) or UniProt Knowledgebase (UniProtKB; http://www.uniprot.org/uniprot/).

### Quantitative PCR

QPCR validations were carried out using the SensiFAST SYBR Lo-ROX kit (Bioline) and first-strand cDNAs as templates. Gene accessions and gene-specific primers are listed in S3 Table. Housekeeping *α-tubulin* and *CAC* genes were used as reference genes for data normalization (data were shown only for *α-tubulin*). PCR conditions were set at 95°C for 2 min, followed by 40 cycles of 95°C for 5 s, and annealing/extension at 60°C for 20 s. Expressions of miRNAs and tasiRNAs were validated using the Mir-X miRNA First-Strand Synthesis and SYBR qRT-PCR kit (Clontech). In a single reaction, sRNA molecules were polyadenylated and reverse transcribed using poly(A) polymerase and SMART MMLV Reverse Transcriptase provided by the kit. List of miRNA and tasiRNA forward primers is provided in S3 Table. U6 small nuclear RNA was used as a reference for data normalization. QPCR conditions were set at 95°C for 10 s, followed by 40 cycles of 95°C for 5 s, and annealing/extension at 60°C for 20 s. Changes of expressions were calculated as normalized fold ratios of three replicates using the ΔΔCT method [32].

### Experimental design and statistical analysis

The experiments were arranged in a completely randomized design with three replications. Data were analyzed using the analysis of variance (ANOVA). Duncan new multiple range test (DNMRT p = 0.05) was used for comparison of the means.

## Results

### Methylation profile in *7B-1* seedlings in response to different lights

**Fig 1:**
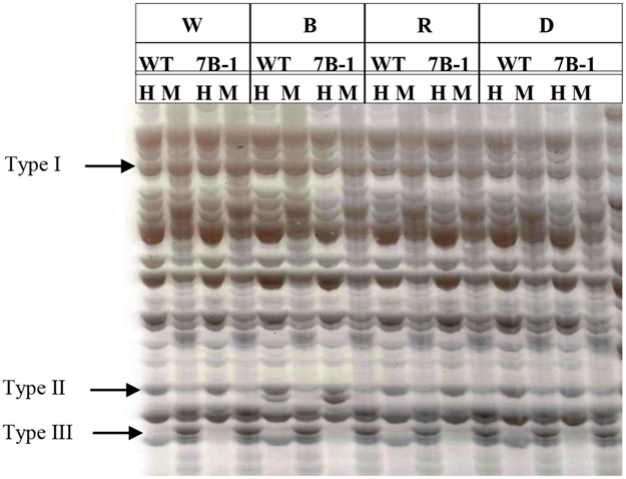
A representative MSAP gel using E2/H4 primer combination. W, B, R, and D correspond to white, blue, and red lights and dark, respectively. Letters H and M show *Eco*RI/*Hpa*II and *Eco*RI/*Msp*I enzymes combinations, respectively. Type I, II and III methylation profiles are indicated by arrows.

Isoschizomers *Hpa*II and *Msp*I display differential sensitivity to DNA methylation as presented in Table 1. MSAP bands could be divided into four types based on their restriction patterns as illustrated in Fig 1. Type I bands, present in the gel for both enzyme combinations, which indicates presence of unmethylated C; type II bands, present only for *Eco*RI/*Hpa*II (hemi-methylation); type III bands, present only for *Eco*RI/*Msp*I (internal full methylation); and type IV bands, absent from both enzyme combinations (external full methylation). Methylation profiles in *7B-1* and WT seedlings were analyzed in W, B, R, and D using a MSAP assay. Twenty four primer combinations were used in our study (S1 Table) and a total of 20 polymorphic bands were identified from the gels and sequenced (Table 2). Several fragments encoded proteins with known regulatory functions, including a phosphatase/tensin-like protein, ribonuclease 3 (RTL3), GTPase-activating protein (GAP), cytochrome b6, anthocyanidin synthase, 14-3-3 protein, histone-lysine N-methyltransferase (HMT), ABC transporter, serine/threonine protein kinase (CIPK), S-adenosylmethionine decarboxylase proenzyme (SAMDC), pentatricopeptide repeat-containing (PPR) protein, and poly(A) RNA polymerase. These proteins regulate biological processes, including protein dephosphorylation, RNA processing and polyadenylation, regulation of G proteins, electron and metabolite transport, anthocyanidin and polyamine biosynthesis, protein-binding, histone methylation, and ABA signaling pathway (Table 2). Other sequenced fragments were originated either from uncharacterized genomic regions or encoded uncharacterized putative proteins.

**Table 1:** Methylation patterns of *Hpa*II and *Msp*I digested genomic DNA.

Type	Methylation status	Sensitivity of enzymes		Bands patterns	
		*Hpa*II	*Msp*I	*Eco*RI/*Hpa*II	*Eco*RI/*Msp*I
**I**	CCGG C^5m^CGG	Insensitive	Insensitive	+	+
	GGCC GGCC				
**II**	^5m^CCGG	Insensitive	Sensitive	+	-
	GGCC				
**III**	C^5m^CGG	Sensitive	Insensitive	-	+
	GG^5m^CC				
**IV**	^5m^C^5m^CGG ^5m^CCGG	Sensitive	Sensitive	-	-
	GGC^5m^ C^5m^ GGCC^5m^				

“+”, present; “-”, absent.

**Table 2:** Differentially methylated MSAP fragments in white, blue, and red lights and dark.

Fragments/primer sets	Sizes (bp)	SGN identifiers^a^	Annotations	Biological processes
**A (E1/H1)**	189	SGN-U577737/Solyc01g107750.2.1	Phosphatase and tensin-like A	Dephosphorylation
**C (E1/H3)**	197	SL2.50ch11:16284156..16284322	No annotation	Uncharacterized
**E (E2/H1)**	282	SL2.50ch12:51555718..51555897	No annotation	Uncharacterized
**F1 (E2/H2)**	188	SGN-U581727/Solyc05g041920.2.1	Ribonuclease 3	RNA processing
**F2 (E2/H2)**	195	SGN-U587595/Solyc03g082590.2.1	GTPase-activating protein gyp7-like	Regulation of Rab GTPase activity
**G (E2/H3)**	595	SGN-U106533/Solyc01g007530.2.1	Cytochrome b6	Electron carrier activity
**H (E2/H4)**	296	SGN-U564994/Solyc10g076670.1.1	Anthocyanidin synthase	Oxidation-reduction process
**I (E2/H5)**	267	SGN-U590882/Solyc05g012420.2.1	14-3-3 protein	Phosphoserine binding
**J (E3/H2)**	222	SGN-U599942/Solyc07g008460.2	Histone-lysine N-methyltransferase	Histone methylation
**K (E3/H3)**	282	SGN-U280616	No annotation	Uncharacterized
**L (E3/H4)**	346	SGN-U597015	No annotation	Uncharacterized
**M (E4/H1)**	300	SGN-U427718	ABC transporter G family member 23	Transport
**N (E4/H2)**	339	SL2.50ch02:50826211..50826511	No annotation	Uncharacterized
**O (E4/H3)**	256	SGN-U569235 /Solyc09g083090.2.1	CBL-interacting serine/threonine kinase 7	Protein phosphorylation
**P1 (E4/H4)**	349	SL2.50ch02:48999344..48999658	No annotation	Uncharacterized
**P2 (E4/H4)**	157	SL2.50ch01:28023377..28023503	No annotation	Uncharacterized
**Q (E5/H1)**	215	SGN-U294267	No annotation	Uncharacterized
**R1 (E5/H2)**	164	SGN-U604807/Solyc06g054460.1	S-adenosylmethionine decarboxylase proenzyme	Polyamine biosynthesis
**R2 (E5/H2)**	193	SGN-U581736/Solyc03g111650.2.1	Pentatricopeptide repeat-containing protein	Uncharacterized
**T (E5/H4)**	123	SL2.50ch09:19095576..19123606	No annotation	Uncharacterized
**V (E6/H2)**	200	SGN-U574172/Solyc01g094000.2	Poly(A) RNA polymerase	Polyadenylation

^a^SGN identifiers include the Unigene IDs/gene names for protein-coding transcripts where applicable, otherwise genomic locations are listed for non-coding transcripts.

The fragments were differentially methylated either in different lights (including D) or in between *7B-1* and WT seedlings (S4 Table). *Phosphatase/tensin*, *HMT* and *14-3-3* were ummethylated in WT and fully methylated (type III) in *7B-1* in all lights. *RTL3* and *cytochrome b6* were both unmethylated in WT in all lights and in *7B-1* only in R, but fully methylated (type III) in *7B-1* in other lights. *GAP*, *anthocyanidin synthase*, and *poly(A) RNA polymerase* were fully methylated (type IV) in WT, but unmethylated in *7B-1* in all lights. *ABC transporter* was fully methylated (type III) in WT in W, B, and D, but unmethylated in R as well as in *7B-1* in all lights. *CIPK* was fully methylated (type IV) in WT in B, R, and W, but unmethylated in D. In *7B-1*, it was fully methylated (type III) in W, B, and R, but unmethylated in D. *SAMDC* and *PPR* were unmethylated in WT and hemi-methylated in *7B-1* in all lights. The results suggested active epigenetic responses to different lights in *7B-1*, which were clearly distinct from those in WT.

### Methylation profile in *7B-1* seedlings in response to different stresses

Methylation profiles in response to exogenous ABA and mannitol-induced stresses in B and D were analyzed in *7B-1* and WT seedlings. A total of 19 polymorphic bands were identified from the gels and sequenced (Table 3). Among those, several sequences encoded stress or defense-related proteins, which have been previously characterized in plants, including SNF4, UDP-glucose flavonoid 3-O-glucosyltransferase (UF3GT), galactinol synthase (GolS), Pti4, N-resistance protein, and MYB transcription factor (AIM1). These fragments were differentially methylated in different light/stress conditions either in WT or *7B-1* or in between (S5 Table). *SNF4* was hemi-methylated in D/MS in both WT and *7B-1*, but fully methylated (IV) in WT and *7B-1* in other light/stress conditions. *UF3GT* was fully methylated (type IV) in WT in B and D by mannitol, but hemi-methylated in both WT and *7B-1* in other light/stress conditions. *GolS* was fully methylated (type IV) in WT and *7B-1* in B/MS and D/MS, while hemi-methylated in WT and *7B-1* in other light/stress conditions. *Pti4* was fully methylated (type IV) in WT in B by mannitol, while hemi-methylated in other conditions. In *7B-1*, it was fully methylated (type IV) in all conditions. *N* gene was fully methylated (type IV) in WT in D by mannitol, but unmethylated in other conditions. In *7B-1*, it was unmethylated in B/MS and D/MS, but fully methylated (type III) in other conditions. *AIM1* was fully methylated (type IV) in WT in all conditions. In *7B-1*, it was fully methylated (type IV) in B/MS and D/MS, but unmethylated in other conditions. Similar to experiments with the lights, these results also indicated an active DNA methylation reprogramming in response to stresses in B and D with distinctive methylation patterns between *7B-1* and WT.

**Table 3:** Differentially methylated MSAP fragments in response to ABA and mannitol in blue light and dark.

Fragments/primer sets	Size (bp)	SGN identifiers^a^	Annotations	Biological processes
**B (E1/H2)**	225	SGN-U570799/Solyc02g068390.2.1	Mitochondrial inner membrane protease subunit 1	Proteolysis
**C (E1/H3)**	230	SGN-U592165	No annotation	Uncharacterized
**D (E1/H4)**	172	SGN-U602058/Solyc10g008270.2	Helix-loop-helix DNA-binding	Uncharacterized
**E (E2/H1)**	213	SL2.50ch09:5926252..5926437	No annotation	Uncharacterized
**F (E2/H2)**	126	SGN-U352649/Solyc11g021170.1.1	ORF82c chloroplast gene	Uncharacterized
**G1 (E2/H3)**	134	SGN-U585007/Solyc06g068160.2.1	SNF4 gene	Stress response
**G2 (E2/H3)**	236	SGN-U585778/Solyc02g081690.1.1	UDP-glucose flavonoid 3-O-glucosyltransferase 6	Flavonoid biosynthesis
**H (E2/H4)**	128	SGN-U563763/Solyc02g062590.2	Galactinol synthase	Stress response
**I (E3/H1)**	200	SGN-U572361/Solyc05g052050.1	DNA-binding protein Pti4	Stress/defense response
**J (E3/H2)**	196	SGN-U567130/Solyc02g067070.2.1	Zinc finger domain containing protein	Uncharacterized
**L (E3/H4)**	147	SGN-U581067/Solyc01g005510.2.1	Laccase-2	Uncharacterized
**M (E4/H1)**	250	SGN-U570799/Solyc02g068390.2.1	Mitochondrial inner membrane protease subunit 1	Proteolysis
**N (E4/H2)**	227	SGN-U481914	No annotation	Uncharacterized
**O (E4/H3)**	179	SL2.50ch10:34406896..34407048	No annotation	Uncharacterized
**P (E4/H4)**	157	SL2.50ch01:28023377..28023503	No annotation	Uncharacterized
**Q (E5/H1)**	128	SGN-U569625/Solyc12g019410.1.1	Kinase family protein	Protein phosphorylation
**R (E5/H2)**	114	SL2.50ch04:28325542..28325629	No annotation	Uncharacterized
**S (E5/H3)**	133	SGN-U591308/Solyc06g083340.2.1	Wound-induced basic protein	Uncharacterized
**U (E6/H1)**	572	SGN-U576251/Solyc12g099120.1.1	ABA-induced MYB transcription factor	Stress response

^a^SGN identifiers include the Unigene IDs/gene names for protein-coding transcripts where applicable, otherwise genomic locations are listed for non-coding transcripts.

### DNA methylation dynamic in *7B-1* in response to different lights and stresses

**Fig 2:**
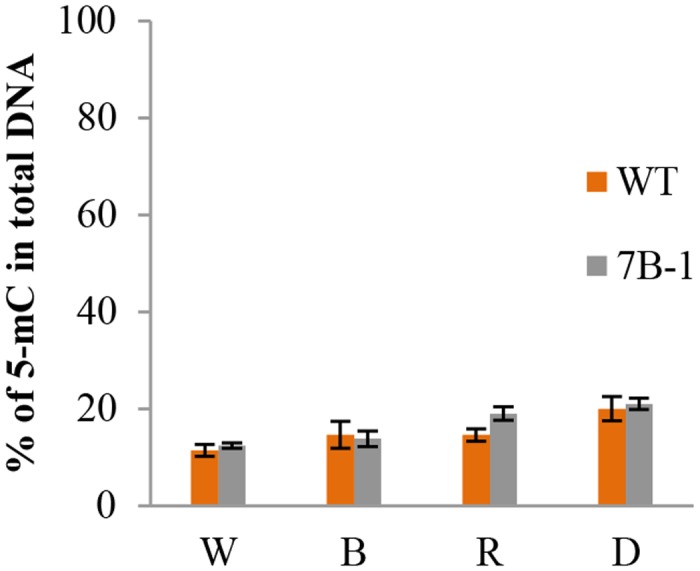
Global genomic methylation levels in *7B-1* and WT in different lights. W, B, R, and D correspond to white, blue, and red lights and dark, respectively. Methylation values were calculated as percentage of the methylated-C in total genomic DNA. Error bars represent standard errors of three technical replicates based on DMNRT (p = 0.05).

Fig 2 shows global changes of genomic methylation level in *7B-1* and WT seedlings in W, B, R, and D. Methylation values were calculated as percentage of the methylated-C in total genomic DNA. Even though there were minor variations of methylation level in different lights and a higher methylation level was observed in D in both *7B-1* and WT and in R in *7B-1* seedlings, yet genomic DNAs did not undergo an extreme methylation changes. Nevertheless, these observation suggested that there might be a dedicated epigenomic mechanism for fine-tune regulation of gene expression in D and R distinctive from those in W and B, as implied by the higher methylation levels in D and R.

**Fig 3:**
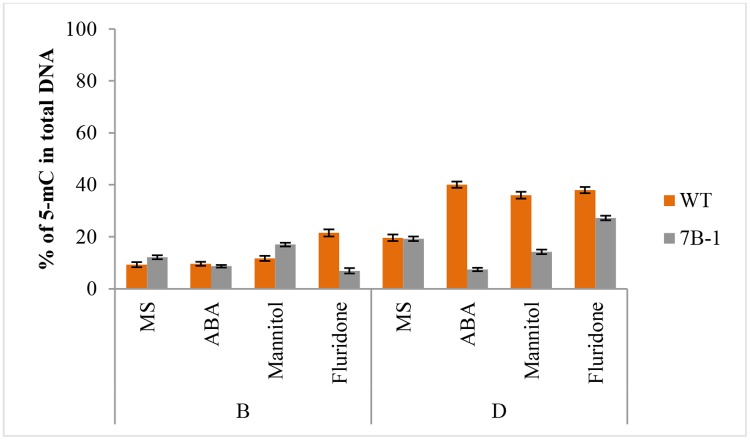
Global genomic methylation changes in *7B-1* and WT in response to ABA, mannitol, and fluridone in blue light (B) and dark (D). Methylation values were calculated as percentage of the methylated-C in total genomic DNA. Error bars represent standard errors of three technical replicates based on DMNRT (p = 0.05).

Global changes of genomic methylation level were also measured in *7B-1* and WT seedlings in response to exogenous ABA, mannitol, and fluridone in B and D (Fig 3). In WT, methylation level was not changed by ABA and mannitol in B, but increased by fluridone. Methylation level in D was higher than B in untreated WT and significantly increased in response to ABA, mannitol and fluridone. In *7B-1*, methylation level was decreased in response to ABA and fluridone in B, but slightly increased by mannitol. Methylation level in D was higher than in B in untreated *7B-1* and decreased in response to ABA and mannitol, but increased by fluridone. In B/stresses conditions, methylation changes were not much significant between *7B-1* and WT, except for the fluridone, however these changes were strikingly prominent in D/stresses conditions, as *7B-1* had a much lower methylation level in general compared to WT. These results indicated extensive changes of genomic methylation landmarks in response to stresses in both light (as the case of B) and dark, which were also distinctively different between *7B-1* and WT.

### Transcriptomic profiling in *7B-1* seedlings in blue light and in response to stresses

Transcriptomic changes between *7B-1* and WT in B and D and also in response to exogenous ABA and mannitol were analyzed using a cDNA-AFLP assay. A total of 19 differentially regulated bands were isolated from the gel and sequenced (Table 4). Several sequences encoded proteins with known regulatory functions, including pectate lyase, transmembrane protein, phosphoserine phosphatase, lipase, RNA helicase, protein phosphatase 2C (PP2C), protein kinase EXS, H^+^-ATPase, and WRKY transcription factor. These proteins are involved in regulation of processes, including cell wall modification, membrane transport, amino acid and lipid biosynthesis, stress response, microsporogenesis, ATP biosynthesis, and regulation of transcription (Table 4). These fragments were differentially regulated between WT and *7B-1* in B or D (in no stress condition) or in response to stresses (S6 Table; comparisons were made based on the intensity of the bands in WT in no stress condition as a reference). In no stress condition, *pectate lyase*, *lipase*, *kinase*, and *H* ^*+*^ *-ATPase* were down regulated in *7B-1* in B. *Phosphoserine phosphatase* and *RNA helicase* genes were also down regulated in *7B-1* but in D. Several other uncharacterized sequences were also differentially regulated between *7B-1* and WT in B and D (S6 Table). In response to stresses, *pectate lyase* and the gene encoded transmembrane protein were both down regulated in *7B-1* in B by mannitol, but not affected by ABA. In D, expression of both genes remained unchanged. *Phosphoserine phosphatase* was not affected by any stresses in B, but down regulated in both *7B-1* and WT in D by ABA, but not mannitol. *Lipase* was down regulated in *7B-1* in B by ABA and mannitol. In D, it was down regulated in both *7B-1* and WT by mannitol. *RNA helicase* was up regulated in *7B-1* in B, but down regulated in D by both ABA and mannitol. *PP2C* was up regulated in *7B-1* in B by ABA and mannitol, while it was down regulated in both *7B-1* and WT in D by mannitol. *Kinase EXS* was up regulated in *7B-1* in B by ABA and mannitol, but not in D. *H* ^*+*^ *-ATPase* was down regulated in WT and *7B-1* in B and D by ABA and mannitol. *WRKY* was up regulated in *7B-1* in B and D by ABA and mannitol, but not in WT.

**Table 4:** Differentially regulated cDNA-AFLP fragments in response to ABA and mannitol in blue light and dark.

Fragments/primer sets	Size (bp)	SGN identifiers^a^	Annotations	Biological processes
**A1 (E1/M1)**	250	SGN-U596469	No annotation	Uncharacterized
**A2 (E1/M1)**	167	SL2.50ch08:16663958..39626043	No annotation	Uncharacterized
**B (E1/M2)**	200	SL2.50ch12:53788392..53788584	No annotation	Uncharacterized
**C (E1/M3)**	107	SGN-U567470/Solyc01g010430.2.1	No annotation	Uncharacterized
**D (E1/M4)**	155	SGN-U585243/Solyc06g083580.2.1	Pectate lyase 1–27	Cell wall modification
**E (E2/M1)**	116	SL2.50ch05:16866694..16867047	No annotation	Uncharacterized
**F (E2/M2)**	165	SL2.50ch08:16663958..39626043	No annotation	Uncharacterized
**G1 (E2/M3)**	323	SL2.50ch10:23440792..23441090	No annotation	Uncharacterized
**G2 (E2/M3)**	131	SGN-U500876/Solyc08g006820.2.1	Transmembrane 9 superfamily member 4	Protein binding
**H (E2/M4)**	87	SGN-U563322/Solyc06g076510.2.1	Phosphoserine phosphatase	Amino-acid biosynthesis
**I1 (E3/M1)**	273	Solyc11g010620.1.1	No annotation	Uncharacterized
**I2 (E3/M1)**	154	SL2.50ch04:63673784..63673909	No annotation	Uncharacterized
**J (E3/M2)**	76	SGN-U566899/Solyc09g0563502.1	Lipase class 3 family protein	Lipid metabolic process
**L (E3/M4)**	316	SGN-U581782/Solyc012g098700.1.1	DEAD-box ATP-dependent RNA helicase 42	Stress response
**N (E4/M2)**	281	SL2.50ch02:37701302..37701557	No annotation	Uncharacterized
**O (E4/M3)**	162	SGN-U566843/Solyc06g051940.2.1	Protein phosphatase 2C	Stress response
**P (E4/M4)**	156	SGN-U576606/Solyc04g071870.1.1	Leucine-rich repeat receptor protein kinase EXS	Microsporogenesis
**Q (E5/M1)**	249	SGN-U574344/Solyc07g017780.2.1	ATPase 8, plasma membrane type	ATP biosynthetic process
**R (E5/M2)**	787	SGN-U570041/Solyc02g088340.2.1	WRKY	Regulation of transcription

^a^SGN identifiers include the Unigene IDs/gene names for protein-coding transcripts where applicable, otherwise genomic locations are listed for non-coding transcripts.

**Fig 4:**
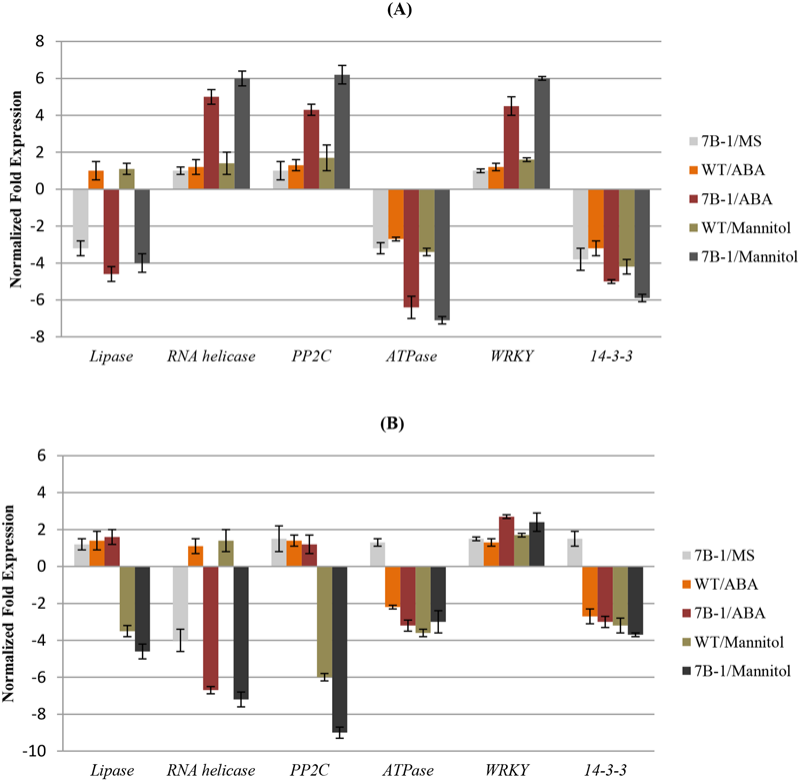
QRT-PCR validation of *lipase*, *RNA helicase*, *PP2C*, *H* ^*+*^ *-ATPase*, *WRKY*, and *14-3-3* genes. Panels A and B show gene expressions in blue light and dark, respectively. Expression changes are presented as normalized fold changes between the test tissues and reference tissue (WT/MS) in corresponding B and D. Positive and negative values indicate up and down regulations of the gene expression, respectively. Twofold threshold was considered as a cutoff value for significant changes in the expression. Error bars represent standard errors of three technical replicates based on DMNRT (p = 0.05).

Expressions of *lipase*, *RNA helicase*, *PP2C*, *H* ^*+*^ *-ATPase*, and *WRKY* genes were further validated using qRT-PCR (Fig 4), and data were in good agreements with the observation from the gel. In addition, expression of a gene encoding 14-3-3 protein was also analyzed using qRT-PCR. *14-3-3* was down regulated in *7B-1* in B in no stress condition, but not in D. In response to ABA and mannitol, it was down regulated in *7B-1* and WT in B as well as D. Transcriptomic analysis in our study revealed that light and stress-induced regulation of gene expression in *7B-1* and WT are different at least to some extents as implied by differential regulation of the above subset of genes.

### DNA methylation and transcriptomic profiling in *7B-1* seedlings in response to 5-azaC

**Fig 5:**
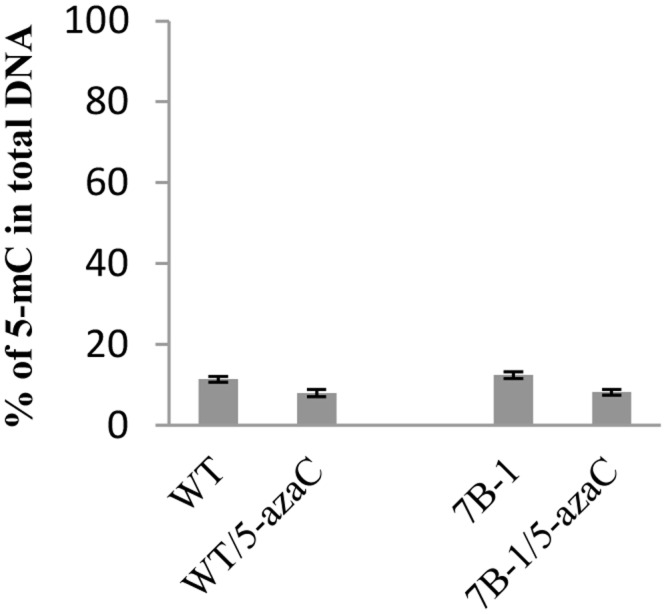
Global genomic DNA methylation changes in *7B-1* and WT seedlings grown in long days in response to 5-azaC treatment. Error bars represent standard errors of three technical replicates based on DMNRT (p = 0.05).

**Fig 6:**
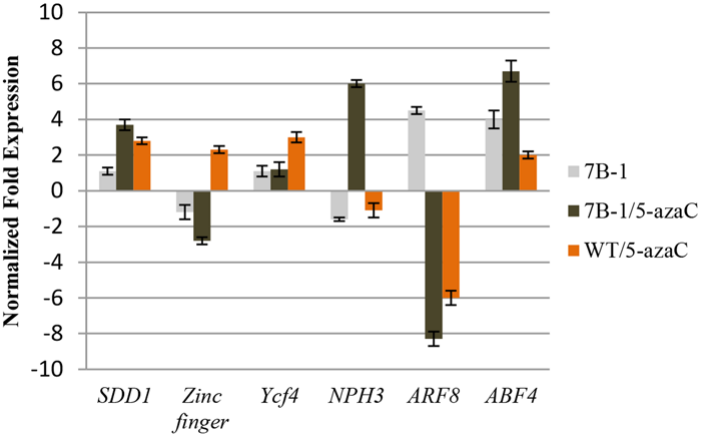
QRT-PCR validation of the differentially regulated genes in response to 5-azaC treatment. Expression changes are presented as normalized fold changes between the test tissues and reference tissue (untreated WT). Positive and negative values indicate up and down regulations of the gene expression, respectively. Twofold threshold was considered as a cutoff value for significant changes in the expression. Error bars represent standard errors of three technical replicates based on DMNRT (p = 0.05).

5-azaC induces aberrant DNA demethylation and genome-wide transcriptional reactivation of silenced genes [28,33]. Global changes of the genomic methylation level in *7B-1* and WT seedlings (2-month old seedlings grown in LD) were measured in response to 5-azaC (Fig 5). Methylation level was relatively similar in untreated *7B-1* and WT seedlings; however in response to 5-azaC, methylation level was reduced (hypomethylation) in *7B-1* and WT of about 1.5 and 1.4 folds, respectively. Some morphological changes were also associated with the treatment, including inhibition of stem elongation in WT and more predominantly in *7B-1* (S1 Fig), and promotion of the lateral shoot formation in both *7B-1* and WT. Structure of the flowers was not affected by 5-azaC and male fertility was not restored in *7B-1*. To investigate the effects of aberrant DNA demethylation on transcriptional regulation of gene expression, transcriptomic changes in 5-azaC-treated *7B-1* and WT seedlings were profiled using a cDNA-AFLP assay. Total of 14 differentially regulated bands were isolated from the gels and sequenced (Table 5). Among those, six transcripts encoded proteins, which have been previously characterized from plants, including subtilisin-like protease (SDD1), CCCH-Type zinc finger, Ycf4, NPH3-type protein, auxin response factor 8 (ARF8), and ABA responsive transcription factor (ABF4). These proteins regulate plant processes, including stomatal morphogenesis, photosynthesis, response to light and hormones (Table 5). Expressions of these genes were validated using qRT-PCR (Fig 6). *SDD1* was up regulated in both *7B-1* and WT seedlings in response to 5-azaC. *Zinc finger* was down regulated in *7B-1*, but slightly up regulated in WT by 5-azaC. Expression of *Ycf4* was not affected in *7B-1*, but increased in WT by 5-azaC. *NPH3* was strongly up regulated in *7B-1* by 5-azaC, but not in WT. *ARF8* expression was higher in untreated *7B-1* seedlings as compared to WT; however it was down regulated strongly in both *7B-1* and WT by 5-azaC. *ABF4* transcripts were found more abundantly in untreated *7B-1* compared to WT. In response to 5-azaC, *ABF4* was strongly up regulated in both *7B-1* and WT. These results indicated that changes of the gene expression in response to 5-azaC were not similar between *7B-1* and WT as indicated by differential regulation of the above subset genes.

**Table 5:** Differentially regulated cDNA-AFLP fragments in response to 5-azaC treatment.

Fragments/primer sets	Size (bp)	SGN identifiers^a^	Annotations	Biological processes	Expression profiles^b^	
					WT/AZA	*7B-1*/AZA
**B (E1/H2)**	308	SL2.50ch09:42398104..42398383	No annotation	Uncharacterized	-	+
**C (E1/H3)**	218	SL2.50ch12:5378839..53788584	No annotation	Uncharacterized	-	+
**D (E1/H4)**	180	Solyc08g079870.1.1	Subtilisin protease	Stomatal morphogenesis	+	+
**E (E2/H1)**	403	Solyc01g088100.2.1	Zinc finger CCCH TYPE protein 22	Transcription regulation	+	-
**F (E2/H2)**	232	SL2.50ch02:43077299..43077478	No annotation	Uncharacterized	+	-
**G (E2/H3)**	190	SL2.50ch05:54038161..54038318	No annotation	Uncharacterized	0	+
**H (E2/H4)**	151	Solyc01g007360.2.1	Photosystem I assembly protein Ycf4	Photosynthesis	+	0
**J (E3/H2)**	254	SL2.50ch03:49354761..49354537	No annotation	Uncharacterized	0	+
**L (E3/H4)**	155	SL2.50ch09:58962162..58962036	No annotation	Uncharacterized	-	+
**M (E4/H1)**	222	SL2.50ch10:45562251..45562058	No annotation	Uncharacterized	-	+
**N (E4/H2)**	256	Solyc04g082920.2.1	Chlorophyl II a-b binding protein	Photosynthesis	-	+
**O (E4/H3)**	205	Solyc09g007820.1.1	Phototropic-responsive NPH3-type protein	Response to light	0	+
**Q (E5/H1)**	302	Solyc02g037530.2.1	Auxin response factor 8	Auxin response	-	-
**S (E5/H3)**	236	Solyc11g044560.1.1	ABA responsive transcription factor	ABA response	0	+

^a^SGN identifiers include the Unigene IDs/gene names for protein-coding transcripts where applicable, otherwise genomic locations are listed for non-coding transcripts.

^b^“0” means no change of expression in response to 5-azaC compared to the reference, “+” means up regulation and “-”means down regulation. Expression profiles are based on the intensities of the bands in 5-azaC-treated tissues as compared to their corresponding untreated tissues.

**Fig 7:**
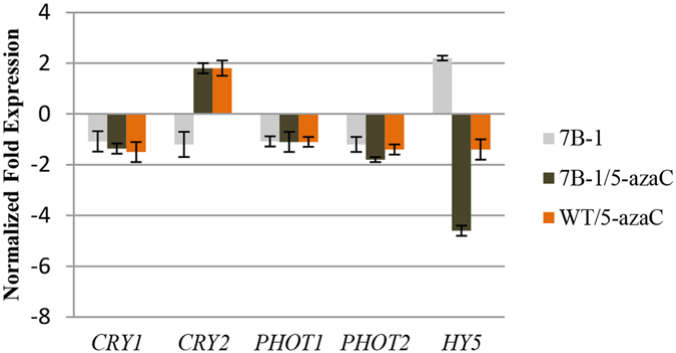
QRT-PCR validation of *CRY1*, *CRY2*, *PHOT1*, *PHOT2*, *and HY5* genes in *7B-1* and WT seedlings in response to 5-azaC. Expression changes are presented as normalized fold changes between the test tissues and reference tissue (untreated WT). Positive and negative values indicate up and down regulations of the gene expression, respectively. Twofold threshold was considered as a cutoff value for significant changes in the expression. Error bars represent standard errors of three technical replicates based on DMNRT (p = 0.05).

In addition to the above genes, expressions of *CRY1/2*, *PHOT1/2*, and *elongated hypocotyl 5* (*HY5*) were also analyzed in 5-azaC-treated *7B-1* and WT seedlings (Fig 7). Expressions of *CRY1*/*2*, and *PHOT1*/*2* were similar between *7B-1* and WT seedlings, but *HY5* expression was higher in *7B-1*. Despite some minor changes (less than 2-fold changes), expressions of *CRY1*/*2*, and *PHOT1*/*2* were not significantly affected by 5-azaC in *7B-1* and WT, while *HY5* strongly down regulated in *7B-1*, but not WT. These results indicated that blue light receptors were not affected by the induced DNA hypomethylation and 5-azaC-induced inhibition of hypocotyl elongation in *7B-1* is independent and not regulated by *CRY1*/*2* and *HY5*.

### Mir167 and mir390 regulate auxin response factors in response to 5-azaC

**Fig 8:**
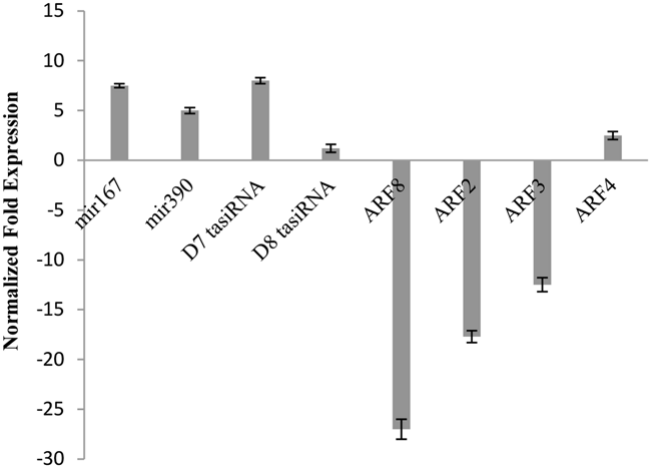
QRT-PCR validation of miRNAs, tasiRNAs, and *ARF*s in 5-azaC-treated *7B-1* seedlings. Expression changes are presented as normalized fold changes between the test tissues and reference tissue (untreated *7B-1*). Positive and negative values indicate up and down regulations of the gene expression, respectively. Twofold threshold was considered as a cutoff value for significant changes in the expression. Error bars represent standard errors of three technical replicates based on DMNRT (p = 0.05).

Mir167 cleaves *ARF8* transcripts and mir390 triggers the production of tasiRNAs from TAS3 mRNA, which in turns cleave and down regulate *ARF2*, *3*, *and 4* [34]. To investigate if these miRNAs were linked to auxin signaling pathway in response to 5-azaC, expressions of mir167, mir390, TAS3-derived tasiRNAs (D7 and D8) and their target *ARF* transcripts (*ARF2/3/4/8*) were analyzed in 5-azaC-treated *7B-1* seedlings using qRT-PCR (Fig 8). Mir167, mir390, D7 tasiRNA were all strongly up regulated in response to 5-azaC, while D8 tasiRNA expression remained unaffected. Primary transcripts of *ARF2*, *3*, *4*, *and 8* were found more abundantly in untreated *7B-1* seedling compared to WT (S2 Fig). Interestingly, *ARF2*, *3*, and *8* were all strongly down regulated in response to 5-azaC, while *ARF4* was slightly up regulated (Fig 8). Expression of sRNAs and/or *ARFs* could have been independently modulated by either DNA hypomethylation or each other in a feedback response or as a result of likely changes of the auxin gradient due to 5-azaC.

## Discussion

The main goal of our study was to understand and highlight the differences in DNA methylation dynamic and transcriptional regulation in response to different lights, stresses and 5-azaC-induced DNA hypomethylation between *7B-1* and WT. In light experiments, methylation changes were mainly displayed in different lights in *7B-1* or WT or in between. Genes with different regulatory functions, including protein dephosphorylation, RNA processing and polyadenylation, regulation of G proteins, electron/metabolite transport, anthocyanidin and polyamine biosynthesis, histone methylation, ABA signaling, and defense response were differentially methylated by lights; however, we did not find any of the key receptors/regulators of light response in our analysis. Among the sequences noteworthy here were *RTL3*, *poly(A) RNA polymerease*, and *histone methyltransferase*, which the first two encode RNA processing enzymes and third one catalyzes histone methylation. Histone methylation and alternative RNA polyadenylation have been shown to be strongly correlated with light regulation of the gene expression in plants [35–38]. However, it’s not clear if these enzymes themselves could also be regulated by DNA methylation. These findings suggested that DNA methylation represented a layer of epigenetic control over the light-induced regulation of gene expression. However, it is unclear if the differences of DNA methylation patterns between *7B-1* and WT are solely due to different lights or could be the primary effect of the *7B-1* mutation, introducing new methylation marks independent of lights.

As MSAP assay does not have the potential to provide a global overview of the methylation dynamic, global methylation changes in different lights were measured in *7B-1* and WT seedlings. Despite some notable variations, genomic DNA did not go through extreme methylation changes in different lights. The data suggested that epigenetic response to different lights in *7B-1* does not encompass extreme methylation reprogramming; however, there could be specialized epigenetic responses for fine-tune regulation of the gene expression in different lights as higher methylation levels was observed in D and R compared those in W and B. In response to ABA and mannitol, several stress-related genes, including *SNF4*, *Pti4*, *N* resistance gene, and *MYB* were identified, which were differentially methylated between *7B-1* and WT. Tobacco plants infected with tobacco mosaic virus showed strong CG hypomethylation at LRR region of the *N* gene [39]. Our results indicated that epigenetic regulation of stress response in *7B-1* and WT requires different DNA methylation reprogramming as indicated by differential methylation of the identified genes. Identification of diverse category of genes with altered DNA methylation patterns in our study provided a clear evidence that firstly, DNA methylation remodeling plays a critical role in plant adaptation to environmental cues, such as light and stresses, and secondly these interactions were different at least party between *7B-1* and WT. Different DNA methylation dynamics were observed in response to ABA, mannitol, and fluridone between *7B-1* and WT in B and D. Changes of DNA methylation level were not so significant between *7B-1* and WT in B in stress conditions, except for the fluridone. On the other hand, these differences were quite remarkable in D, suggesting that DNA hypermethylation in D is an active epigenetic response to stresses in WT. The results indicated that in addition to the crosstalk between light and hormonal signaling pathways, the WT response to stresses in B and D could partially be regulated through an extensive DNA methylation reprogramming. In contrary, *7B-1* response to stresses in B and D does not impose an extreme DNA methylation remodeling, but it’s very likely to be different from those of WT in D. Hypermethylation in D is in good agreement with the slow rate of global transcription in D in general, in contrast to the faster rate of light-induced transcription. Stress-induced increase of methylation level in WT in D could also be explained in a way that hypermethylation further did reduce the global transcription rate in favor of slowing down the energy consumption, while expression of a subset of hypomethylated stress-related genes could likely be expanded to help the cell to cope with the stress. However, it remains unclear if the sudden drop of DNA methylation level in *7B-1* in response to ABA and mannitol in D is due to the direct effects of stresses or as a result of autonomous interplay between *7B-1* mutation and affected hormonal sensitivities in *7B-1*.

Fluridone prevents ABA biosynthesis, but it was not clear if it affected ABA level in *7B-1* and WT equally. Different methylation response of *7B-1* to fluridone in B compared to WT could have been regulated by elevated sensitivity or higher endogenous level of the ABA in *7B-1* seedlings. Not taking the light-ABA interaction into account in D, fluridone increased methylation level similarly in *7B-1* and WT. Interestingly, ABA treatment or inhibition of ABA synthesis did not influence the methylation level in WT in D, but they reduced and induced the methylation level in *7B-1*, respectively.

Using a cDNA-AFLP assay, several transcripts were identified, which were differentially regulated between *7B-1* and WT in B or D (no stress condition). These transcripts encoded regulatory proteins involved in process, such as cell wall modification, lipid and amino acid biosynthesis, microsporogenesis, and ATP biosynthesis. These findings highlighted some of the differences of blue light and dark regulation of gene expression between *7B-1* and WT. *H* ^*+*^ *-ATPase* and *14-3-3* genes were both down regulated in *7B-1* in B. PHOT1/2 mediate B-dependent activation of H^+^-ATPase in guard cells via phosphorylation and subsequent binding of the 14-3-3 proteins [40]. In contrary, ABA could suppress stomatal opening by inhibiting phosphorylation of H^+^-ATPase [41]. Hlavinka et al. [42] reported that B-induced stomatal opening is impaired in *7B-1*, but unlikely to be affected by H^+^-ATPase. Our data suggested that B has positively regulated *H* ^*+*^ *-ATPase* expression in WT, as a defect in B perception in *7B-1* resulted in lower expression of *H* ^*+*^ *-ATPase* transcripts. In addition, B-insensitivity and elevated level of ABA in *7B-1* could have possibly led to partial inactivation of H^+^-ATPase. On the other hand, down regulation of *14-3-3* expression in B could slow down H^+^-ATPase phosphorylation rate, which in turn reduces the B-induced stomatal opening.

In response to ABA and mannitol, several stress-related genes, including *lipase*, *RNA helicase*, *PP2C*, and *WRKY* were differentially regulated between *7B-1* and WT in B or D. Several studies have shown differential expression of *lipases* in response to abiotic stresses [43,44], but the actual function of these genes in plant adaptation to stress is largely unknown. Understating how down regulation of this gene in *7B-1* is connected to stress response requires further functional studies. *RNA helicases* have been implicated in every step of RNA metabolism [45], while their involvement in response to abiotic stress is only beginning to emerge. There are few reports displaying up regulation of the *DEAD-box* family of *RNA helicases* in response to abiotic stress, suggesting that they might play important roles in stabilizing plant growth under stress conditions by regulating stress-induced pathways [46–48]. In pea, two *DEAD-box helicases*, *PDH45* and *PDH47*, were induced by a variety of abiotic stresses [48], while in *Arabidopsis*, two *DEAD-box RNA helicases*, *STRS1* and *STRS2*, negatively regulated the stress response [49]. Up regulation of *RNA helicase* in *7B-1* by ABA and mannitol could be associated with B-specific stress-tolerance of *7B-1*, however down regulation of this gene in D in no stress condition and by ABA and mannitol, suggests an alternative regulatory function for this gene rather than a stress-related one. *PP2Cs* act redundantly in ABA signaling and stress response in plants [50–53]. *PP2Cs* have been reported to be differentially expressed in response to different abiotic stresses [51–53]. Inactivation of PP2Cs by ABA receptors, such as *OsPYL5* and *AtPYL* enhanced drought tolerance in rice and *Arabidopsis*, respectively [51, 52]. Expression of *OsSIPP2C1* was up regulated in rice in response to ABA, drought and salinity [53]. Up regulation of *PP2C* in *7B-1* by ABA and mannitol in B could in part contribute to *7B-1* stress tolerance, but down regulation of this gene in WT and *7B-1* by mannitol in D, suggests a different mode of action for this gene in D as compared to B, which has yet to be understood. Several *WRKY*s have been identified, which positively regulate plant response to a range of abiotic stresses [54–56]. Up regulation of *WRKY* in *7B-1* in response to ABA and mannitol in B and D, could in part mediate the higher tolerance of *7B-1* to these stresses. Response to stress in light and dark is coordinated by a synchronized action of many stress-inducible genes, some being positively regulated while others down regulated [57]. Our findings indicated that blue light and dark regulation of stress response in *7B-1* is modulated at least partly through a different transcriptional reprogramming in comparison to those in WT. These differences could be due to the indirect effect of *7B-1* mutation, which has impaired B signaling and hormonal balance in *7B-1*, altering the downstream gene expression as a result. In addition to the above mentioned protein-coding mRNAs, several non-coding transcripts were also identified in our cDNA-AFLP experiment, which have been differentially expressed in different lights and stresses. These fragments are likely the amplified long non-coding RNAs (lncRNAs), which could have been transcribed from intronic, intergenic, intragenic, promoters, and untranslated regions. Several lncRNAs have been identified from *Arabidopsis* [58], rice [59] and wheat [60], which were suggested to be related to abiotic stress response based on their expression profile. However, lncRNAs are still largely unknown in plants, which makes their analysis rather challenging, therefore we turned our attention to those protein-coding transcripts.

Phenotypic and developmental changes could be induced in plants by aberrant demethylation of DNA [28,33,61]. 5-azaC-induced hypomethylation in *7B-1* (1.5-fold decrease of genomic methylation level) inhibited stem elongation and promoted shoot branching, but did not affect the flower structures and male sterility. Transcriptomic changes were mainly profiled in the stem tissue as induced morphological changes were manifested primarily in this tissue. Among those differentially regulated transcripts, several transcripts encoded regulatory proteins involved in processes, such as stomatal morphogenesis, photosynthesis, and response to light and hormones. These transcripts were differentially regulated in response to 5-azaC in either *7B-1* or WT or in between. These results indicated that induced hypomethylation has affected the transcriptional regulatory network or at least a subset of genes unequally between *7B-1* and WT.

NPH3 (non-phototropic hypocotyl 3) is a phototropin-interacting protein, which is essential for PHOT1-dependent phototropic response [62]. Blue light together with PHOT1 stimulate NPH3 activity via dephosphorylation, but it not clear if B induces the *NPH3* expression [63]. *NPH3* had a lower expression in untreated *7B-1* seedlings compared to WT, and it was up regulated in response to 5-azaC in *7B-1*, but not in WT. Earlier experiments in our lab showed that the phototropism was impaired in *7B-1* seedlings (Bergougnoux and Fellner, unpublished data). This could also be addressed by lower level of *NPH3* transcripts in *7B-1* seedlings, which itself could be due to a tradeoff mechanism between inactive form of the NPH3 protein (phosphorylated) and *NPH3* transcripts level. Up regulation of this gene by 5-azaC in *7B-1* and not WT could possibly be explained by i) the indiscriminate removal of epigenetic marks from *NPH3* exclusively in *7B-1*, which could have induced the *NPH3* expression, or ii) partial restoration of B sensitivity in *7B-1* in response to 5-azaC, which could have balanced off the *NPH3* transcripts. This brings into question if phototropic response in *7B-1* is restored by 5-azaC, which has to be yet verified. *PHOT1*/*2* expressions were similar between *7B-1* and WT, which indicated that the reduced rate of phototropism in *7B-1* was independent and not mediated through its receptors at the transcriptional level. Similar expression of *CRY1*/*2* between *7B-1* and WT also indicated that that the defect in blue light signaling as well as the reduced de-etiolation in *7B-1* were rather independent and not regulated by *CRY1*/*2*, at least at the transcriptional level, suggesting that the *7B-1* mutation had probably affected the downstream components of the light signaling pathway, and not the receptors. HY5 plays a key role in promoting photomorphogenesis by regulating the transcription of a wide range of genes, and its abundance is negatively correlated with hypocotyl elongation [64]. In our study, *HY5* had a higher expression in *7B-1* compared to WT seedlings. Keeping in mind that *7B-1* had a longer hypocotyl than WT, brings the observation to the contrary with the general function of HY5 in inhibition of hypocotyl elongation, which higher abundance of HY5 protein is associated with shorter length of the hypocotyl. However, it should be noted that higher level of *HY5* transcript does not necessarily imply to a higher protein accumulation. Even though, 5-azaC inhibited hypocotyl elongation in *7B-1*, yet down regulation of *HY5* in *7B-1* in response to 5-azaC could not be agreeably tailored to its central role in light-inhibition of hypocotyl elongation. Increasing evidences suggest that hormones, such as ethylene, gibberellins and cytokinins also regulate the accumulation of HY5 [65], therefore primary expression of *HY5* in *7B-1* and in response to 5-azaC could perhaps be in part mediated by hormonal imbalance and altered sensitivity in *7B-1*.

*ARFs* modulate auxin signaling by regulating the expression of auxin-response genes [66]. Mir167 and mir390 are tightly connected to auxin signaling pathway via down regulation of *ARF8* and *ARF2/3/4* transcripts, respectively [34]. Effect of DNA hypomethylation on the crosstalk between miRNAs, *ARFs*, and auxin signaling was investigated in our study. Mir167, mir390, D7 tasiRNA were all up regulated in 5-azaC-treated *7B-1* seedlings, which expectedly down regulated the expression of their target *ARF* transcripts except for *ARF4*; *ARF4* was slightly up regulated. These results pointed to a combinatory interplay between DNA hypomethylation and miRNA modulation of auxin signaling via spatial regulation of *ARFs*. Furthermore, our findings underlined the vital regulatory functions of miRNA in balancing off the gene expression in response to uncommon epigenetic marks, such as induced DNA hypomehtyation. However, it is unclear if the changes of miRNA expressions were due to the direct effect of 5-azaC treatment or modulated through a feedback regulation mediated by auxin gradient in *7B-1* seedlings. We did not measure the auxin level in 5-azaC-treated *7B-1* seedlings, but as auxin transport is required for hypocotyl elongation in light [67], one possible explanation for inhibition of stem elongation in *7B-1* could be that 5-azaC has reduced the rate of auxin synthesis in shoot apical meristem, slowing down its transport to other tissues, which in turn could have negatively regulated the stem elongation. This notion could be coupled by the observations that *ARFs* were down regulated by 5-azaC, but also to mention that the primary transcripts of *ARFs* were found more abundantly in untreated *7B-1* seedlings compared to WT, which could imply a more active auxin signaling/responsive gene expression in *7B-1*. *ARFs* seem to control both auxin sensitivity and homeostasis as reported in *Arabidopsis* [66,68]. Several reports indicated involvement of *ARFs*, such as *ARF2*, *6*, and *8* in regulation of the hypocotyl growth in an auxin-dependent fashion [68–70]. Target genes of *ARF2*, *3*, *and 4* remain largely unknown, thus the mechanisms linking these *ARFs* to context-specific cellular responses are poorly understood. Tian et al. [70] suggested that *ARF8* could control the free level of IAA in a negative feedback by regulating the expression of *GH3* genes. Based on our findings, it could be concluded the defect in blue light signaling in *7B-1* was not mediated by its photoreceptors and the mutation had probably affected downstream components of the light signaling pathway. Differences in the regulation of gene expression in response to lights and stresses between *7B-1* and WT clearly showed that *7B-1* mutation has extensively affected the light- and stress-responsive gene expression in *7B-1*. The mutation also has changed the methylation dynamic in *7B-1* and brought along a whole new set of methylation marks in response to different light and stress scenarios, which could be either the direct effect of the mutation or as an adaptive response to changes of the gene expression portfolio. This information adds on to the fact that the *7B-1* is a complex mutation with its primary effect yet unknown. A probable explanation could be that male-sterility in *7B-1* is governed by interplays between blue light signaling defect and altered level of endogenous hormones, which in turn could have adversely affected the expression of genes involved in anther and pollen development. Comparative studies on light- and stress-responsive epigenomes and transcriptomes will enhance our understanding of plant adaptation to lights and stresses. Overall, our study highlighted some of the differences in epigenetic and transcriptional regulation of light and stress responses between *7B-1* and WT, and shed lights on the crosstalk between DNA hypomethylation and miRNA regulation of *ARFs*.

## Supporting Information

S1 Fig Effect of 5-azaC treatment on *7B-1* stem elongation. (TIF)

S2 Fig QRT-PCR validation of *ARF*s in *7B-1* seedling grown in long days. Expression changes are presented as normalized fold changes between *7B-1* and WT reference tissue. Twofold threshold was considered as a cutoff value for significant changes in the expression. Error bars represent standard errors of three technical replicates based on DMNRT (p = 0.05).
(TIF)

S1 Table Adapter and primer sequences for the MSAP analysis. (DOC)

S2 Table Adapter and primer sequences for cDNA-AFLP analysis. (DOC)

S3 Table List of primers used for qPCR validations. (DOCX)

S4 Table Schematic representation of light-MSAP fragments on the gel. (DOCX)

S5 Table Schematic representation of stress-MSAP fragments on the gel. (DOCX)

S6 Table Schematic representation of the expression of cDNA-AFLP fragments on the gel. (DOCX)

S1 File Genomic methylation and qRT-PCR values. (DOCX)
