# Supplementary figures and images for: DNA Methylation and Transcriptomic Changes in Response to Different Lights and Stresses in 7B-1 Male-Sterile Tomato
Source: PLoS One. 2015 Apr 7;10(4):e0121864. doi: 10.1371/journal.pone.0121864 (PMC4388563; doi:10.1371/journal.pone.0121864)

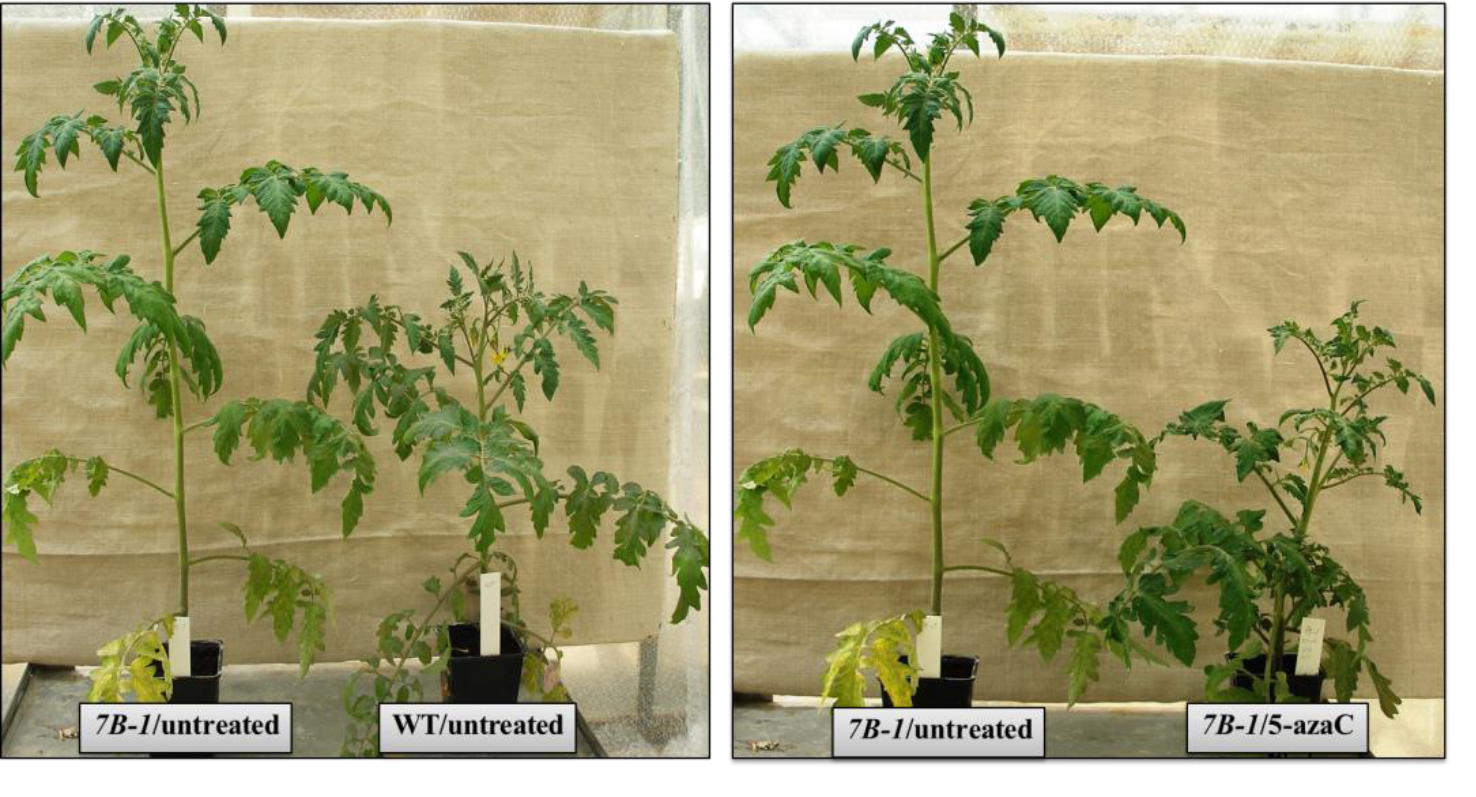

Supplement: S1 Fig — (TIF) [file pone.0121864.s001.tif]

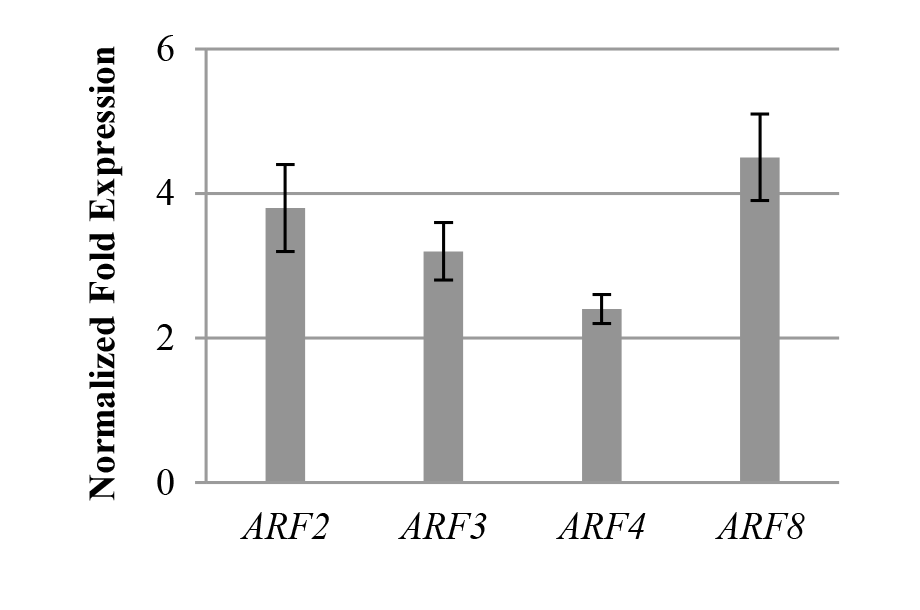

Supplement: S2 Fig — Expression changes are presented as normalized fold changes between 7B-1 and WT reference tissue. Twofold threshold was considered as a cutoff value for significant changes in the expression. Error bars represent standard errors of three technical replicates based on DMNRT (p = 0.05). (TIF) [file pone.0121864.s002.tif]
