# Supplementary material for: DNA Methylation and Transcriptomic Changes in Response to Different Lights and Stresses in 7B-1 Male-Sterile Tomato
Source: PLoS One. 2015 Apr 7;10(4):e0121864. doi: 10.1371/journal.pone.0121864 (PMC4388563; doi:10.1371/journal.pone.0121864)
Supplement: S1 Table — (DOC) [file pone.0121864.s003.doc]

**S1 Table. Adapter and primer sequences for the MSAP analysis.**

| **Adapters and primers** | ***Eco*R I (E)** | ***Hpa* II/*Msp* I (H/M)** |
| --- | --- | --- |
| Adapter1 | 5'-CTCGTAGACTGCGTACC-3' | 5'-GATCATGAGTCCTGCT -3' |
| Adapter2 | 5'-AATTGGTACGCAGTCTAC-3' | 5'-CGAGCAGGACTCATGA -3' |
| Preamp primer | 5'-GACTGCGTACCAATTCA-3'(E1) | 5'-ATCATGAGTCCTGCTCGGT -3'(HM1) |
| Selective primers | E1+AC(E01) | HM1+CG(HM01) |
|  | E1+AG(E02) | HM1+GC(HM02) |
|  | E1+CT(E03) | HM1+GA(HM03) |
|  | E1+TC(E04) | HM1+AG(HM04) |
|  | E1+CC(E05) |  |
|  | E1+CG(E06) |  |
