# Supplementary material for: DNA Methylation and Transcriptomic Changes in Response to Different Lights and Stresses in 7B-1 Male-Sterile Tomato
Source: PLoS One. 2015 Apr 7;10(4):e0121864. doi: 10.1371/journal.pone.0121864 (PMC4388563; doi:10.1371/journal.pone.0121864)
Supplement: S3 Table — (DOCX) [file pone.0121864.s005.docx]

**S3 Table. List of primers used for qPCR validations.**

| **Genes/miRNAs** | **SGN Identifiers/miRBase accessions** | **Forward primers (5'-3')** | **Reverse primers (5'-3')** |
| --- | --- | --- | --- |
| Lipase | Solyc09g056350.2.1 | CTCGACTATGGCGAACAGGT | TCAGCTAAGCGTCTGCGATT |
| RNA helicase | Solyc012g098700.1.1 | TGTTGCCGAGAACGTGGTAA | TTCCAGCAGAGGAGGACTGA |
| PP2C | Solyc06g051940.2.1 | TCGCCTTAACGGTAGTCAGC | CACCGTTTCCTGAATGCTGC |
| ATPase | Solyc07g017780.2.1 | GTTGAATCAATGGGGAGTCATCC | CGGTTTCCTTTTGATCCGCC |
| WRKY | Solyc02g088340.2.1 | TTGACAATTCCACCTCGCCC | GCTTCTACTGGAGCCACCTG |
| 14-3-3 | Solyc01g010360.2.1 | TCCTTTTGGCTTGTAATTTGCAC | GACATGATACGCCACGAAGC |
| SDD1 | Solyc08g079870.1.1 | CTGTTGGTGTTGCCCCTCTT | CTGAGTTATCTACTGAGCCATGGAA |
| Zinc finger | Solyc01g088100.2.1 | TGTGCTTGATAGCTGGGGTG | CGGCCAACTGTTTTCCACAG |
| Ycf4 | Solyc01g007360.2.1 | TGGCCTTTTCTTTGGTGATAGA | CGAATCCGACCCAACATGGA |
| NPH3 | Solyc09g007820.1.1 | TCAAAACGAGAGGCTTCCCC | CTCGGAAACACGTTCCCTCA |
| ABF4 | Solyc11g044560.1.1 | CCAGGGAAAAGGCTTCCCAT | CCGCAGACTCCCTGTTCTTT |
| CRY1 | Solyc04g074180.2.1 | TCCTTTAATGCTGATTTGC | TGGGAGGAAGAAGTGGAGC |
| CRY2 | Solyc09g090100.2.1 | TTGCCCGAGCAGTTCATCTT | CAAGTCACCGTCCACGTTTG |
| PHOT1 | Solyc11g072710.1.1 | GCTGGCAAAGAGAGGGGAAT | CCCGATTGCAATGCTTCTCG |
| PHOT2 | Solyc01g097770.2.1 | GAAGGTGTTAATGAGAAAGAAT | CGTCCTGACTAGACTTTATA |
| HY5 | Solyc08g061130.2.1 | AATGCAAGAGCAAGCGACGA | TGAGTCCCAGCTGATGGTTG |
| ARF8 | Solyc02g037530.2.1 | AATTGGGGCAGATGTTCGGT | CAATCAGTACTCCAGCGATCCA |
| ARF2 | Solyc03g118290.2.1 | AGAGGGTGAGCTCGTGTACT | TCTTGATTTGGCTCCGGCAT |
| ARF3 | Solyc02g077560.2.1 | GGTGGTTTCTCTGTCCCTCG | AGCTCTCCATCACCAGTCCT |
| ARF4 | Solyc11g069190.1.1 | TGATAAACCAGTCCGCCCAC | AGGTTTTCCCCAAGCACCAA |
| α-tubulin | Solyc04g077020.2.1 | TGAGGTCTTCTCACGCATTGACCA | AATCCTTCTCGAGGGCAGCAAGAT |
| CAC | SGN-U314153 | CCTCCGTTGTGATGTAACTGG | ATTGGTGGAAAGTAACATCATCG |
| Mir167 | MIMAT0007917 | TGAAGCTGCCAGCATGATCTA | mRQ 3' primer* |
| Mir390 | MIMAT0035479 | AAGCTCAGGAGGGATAGCG | mRQ 3' primer |
| Tasi-D7 | no entry | TTCTTGACCTTGTAAGACCC | mRQ 3' primer |
| Tasi-D8 | no entry | TTCTTGACCTTGTAAGACCTT | mRQ 3' primer |

* mRQ 3' primer was provided by the Mir-X™ miRNA First-Strand Synthesis and SYBR® qRT-PCR kit (Clontech).
