# Supplementary material for: DNA Methylation and Transcriptomic Changes in Response to Different Lights and Stresses in 7B-1 Male-Sterile Tomato
Source: PLoS One. 2015 Apr 7;10(4):e0121864. doi: 10.1371/journal.pone.0121864 (PMC4388563; doi:10.1371/journal.pone.0121864)
Supplement: S4 Table — (DOCX) [file pone.0121864.s006.docx]

**S4 Table. Schematic representation of light-MSAP fragments on the gel.**

| **Fragments** | **W** | | | | **B** | | | | **R** | | | | **D** | | | |
| --- | --- | --- | --- | --- | --- | --- | --- | --- | --- | --- | --- | --- | --- | --- | --- | --- |
|  | **WT** | | ***7B-1*** | | **WT** | | ***7B-1*** | | **WT** | | ***7B-1*** | | **WT** | | ***7B-1*** | |
|  | **H** | **M** | **H** | **M** | **H** | **M** | **H** | **M** | **H** | **M** | **H** | **M** | **H** | **M** | **H** | **M** |
| A | **-** | **-** |  | **-** | **-** | **-** |  | **-** | **-** | **-** |  | **-** | **-** | **-** |  | **-** |
| C | **-** | **-** |  | **-** | **-** | **-** |  | **-** | **-** | **-** |  | **-** | **-** | **-** |  | **-** |
| E |  | **-** |  | **-** |  | **-** |  | **-** |  | **-** |  | **-** | **-** | **-** | **-** | **-** |
| F1 | **-** | **-** |  | **-** | **-** | **-** |  | **-** | **-** | **-** | **-** | **-** | **-** | **-** |  | **-** |
| F2 |  |  | **-** | **-** |  |  | **-** | **-** |  |  | **-** | **-** |  |  | **-** | **-** |
| G | **-** | **-** |  | **-** | **-** | **-** |  | **-** | **-** | **-** | **-** | **-** | **-** | **-** |  | **-** |
| H |  |  | **-** | **-** |  |  | **-** | **-** |  |  | **-** | **-** |  |  | **-** | **-** |
| I | **-** | **-** |  | **-** | **-** | **-** |  | **-** | **-** | **-** |  | **-** | **-** | **-** |  | **-** |
| J | **-** | **-** |  | **-** | **-** | **-** |  | **-** | **-** | **-** |  | **-** | **-** | **-** |  | **-** |
| K | **-** | **-** |  | **-** | **-** | **-** |  | **-** | **-** | **-** |  | **-** | **-** | **-** |  | **-** |
| L | **-** | **-** |  | **-** | **-** | **-** |  | **-** | **-** | **-** |  | **-** | **-** | **-** |  | **-** |
| M |  | **-** | **-** | **-** |  | **-** | **-** | **-** | **-** | **-** | **-** | **-** |  | **-** | **-** | **-** |
| N |  | **-** | **-** | **-** |  | **-** | **-** | **-** |  | **-** | **-** | **-** |  | **-** | **-** | **-** |
| O |  |  |  | **-** |  |  |  | **-** |  |  |  | **-** | **-** | **-** | **-** | **-** |
| P1 |  |  | **-** | **-** |  |  | **-** | **-** |  |  | **-** | **-** |  |  | **-** | **-** |
| P2 |  |  | **-** |  |  |  | **-** |  |  |  | **-** |  |  |  | **-** |  |
| Q | **-** | **-** |  | **-** | **-** | **-** |  | **-** | **-** | **-** |  | **-** | **-** | **-** | **-** | **-** |
| R1 | **-** | **-** | **-** |  | **-** | **-** | **-** |  | **-** | **-** | **-** |  | **-** | **-** | **-** |  |
| R2 | **-** | **-** | **-** |  | **-** | **-** | **-** |  | **-** | **-** | **-** |  | **-** | **-** | **-** |  |
| T |  | **-** | **-** | **-** |  | **-** | **-** | **-** |  | **-** | **-** | **-** |  | **-** | **-** | **-** |
| V |  |  | **-** | **-** |  |  | **-** | **-** |  |  | **-** | **-** |  |  | **-** | **-** |

“–“ indicates presence of a band in the gel. W, B, R, and D correspond to white, blue, and red lights and dark, respectively. H and M correspond to *EcoR* I/*Hpa* II and *EcoR* I/*Msp*I combinations.
