# Supplementary material for: DNA Methylation and Transcriptomic Changes in Response to Different Lights and Stresses in 7B-1 Male-Sterile Tomato
Source: PLoS One. 2015 Apr 7;10(4):e0121864. doi: 10.1371/journal.pone.0121864 (PMC4388563; doi:10.1371/journal.pone.0121864)
Supplement: S5 Table — (DOCX) [file pone.0121864.s007.docx]

**S5 Table. Schematic representation of stress-MSAP fragments on the gel.**

|  | **B/MS** | | | | **D/MS** | | | | **B/ABA** | | | | **B/Mannitol** | | | | **D/ABA** | | | | **D/Mannitol** | | | |
| --- | --- | --- | --- | --- | --- | --- | --- | --- | --- | --- | --- | --- | --- | --- | --- | --- | --- | --- | --- | --- | --- | --- | --- | --- |
| **Fragments** | **WT** | | ***7B-1*** | | **WT** | | ***7B-1*** | | **WT** | | ***7B-1*** | | **WT** | | ***7B-1*** | | **WT** | | ***7B-1*** | | **WT** | | ***7B-1*** | |
|  | **H** | **M** | **H** | **M** | **H** | **M** | **H** | **M** | **H** | **M** | **H** | **M** | **H** | **M** | **H** | **M** | **H** | **M** | **H** | **M** | **H** | **M** | **H** | **M** |
| B |  | - |  | - |  | - |  | - |  |  |  |  |  |  |  |  |  |  |  |  |  |  |  |  |
| C |  |  |  |  |  |  |  |  |  | - |  | - |  |  |  | - |  |  |  | - |  |  |  | - |
| D | - |  | - |  | - |  | - |  | - |  | - |  |  |  | - |  | - |  | - |  | - |  | - |  |
| E |  |  |  |  |  |  |  |  |  |  |  |  |  |  |  |  |  |  |  |  |  |  |  |  |
| F | - | - |  | - | - | - |  | - |  | - |  | - |  | - |  | - | - | - |  | - | - | - |  | - |
| G1 |  |  |  |  | - |  | - |  |  |  |  |  |  |  |  |  |  |  |  |  |  |  |  |  |
| G2 | - |  | - |  | - |  | - |  | - |  | - |  |  |  | - |  | - |  | - |  |  |  | - |  |
| H |  |  |  |  |  |  |  |  | - |  | - |  | - |  | - |  | - |  | - |  | - |  | - |  |
| I | - |  |  |  | - |  |  |  | - |  |  |  |  |  |  |  | - |  |  |  | - |  |  |  |
| J |  |  |  |  |  |  |  |  |  |  |  |  |  |  |  |  |  |  |  |  |  |  |  |  |
| L |  |  |  |  | - |  | - |  |  |  |  |  |  |  |  |  |  |  |  |  |  |  |  |  |
| M | - |  | - |  | - |  | - |  | - |  | - |  |  |  | - |  | - |  | - |  | - |  | - |  |
| N |  |  |  |  |  |  |  |  |  |  |  |  |  |  | - |  |  |  | - |  |  |  |  |  |
| O |  |  |  |  | - |  | - |  |  |  |  |  |  |  |  |  |  |  |  |  |  |  |  |  |
| P | - | - | - | - | - | - | - | - | - | - |  | - | - | - |  | - | - | - |  | - |  |  |  | - |
| Q |  |  | - |  |  |  | - |  |  |  | - |  |  |  | - |  |  |  | - |  |  |  |  |  |
| R |  | - |  |  |  |  |  | - |  |  |  | - |  |  |  | - |  |  |  | - |  |  |  | - |
| S |  |  |  |  | - |  | - |  |  |  |  |  |  |  |  |  |  |  |  |  |  |  |  |  |
| U |  |  |  |  |  |  |  |  |  |  | - | - |  |  | - | - |  |  | - | - |  |  | - | - |

“–“ indicates presence of a band in the gel. H and M correspond to *EcoR* I/*Hpa* II and *EcoR* I/*Msp*I combinations.
