# Supplementary material for: DNA Methylation and Transcriptomic Changes in Response to Different Lights and Stresses in 7B-1 Male-Sterile Tomato
Source: PLoS One. 2015 Apr 7;10(4):e0121864. doi: 10.1371/journal.pone.0121864 (PMC4388563; doi:10.1371/journal.pone.0121864)
Supplement: S6 Table — (DOCX) [file pone.0121864.s008.docx]

**S6 Table. Schematic representation of the expression of cDNA-AFLP fragments on the gel.**

| **Fragments** | **B/MS** | | **B/ABA** | | **B/mannitol** | | **D/MS** | | **D/ABA** | | **D/mannitol** | |
| --- | --- | --- | --- | --- | --- | --- | --- | --- | --- | --- | --- | --- |
|  | ***7B-1*** | **WT** | ***7B-1*** | **WT** | ***7B-1*** | **WT** | ***7B-1*** | **WT** | ***7B-1*** | **WT** | ***7B-1*** | **WT** |
| A1 | - | C | - | = | = | = | = | C | - | - | - | - |
| A2 | = | C | = | + | = | = | = | C | - | - | - | - |
| B | = | C | = | = | = | = | = | C | = | = | - | - |
| C | - | C | - | = | - | - | = | C | = | = | - | - |
| D | - | C | = | = | - | - | = | C | = | = | = | = |
| E | - | C | - | = | - | - | = | C | = | = | - | - |
| F | - | C | - | = | - | = | = | C | = | = | - | - |
| G1 | - | C | - | = | - | - | = | C | - | = | = | - |
| G2 | = | C | = | = | - | = | = | C | = | = | = | = |
| H | = | C | = | = | = | = | - | C | - | - | = | = |
| I1 | = | C | = | = | = | = | = | C | = | = | = | + |
| I2 | = | C | + | + | + | = | = | C | + | + | = | = |
| J | - | C | - | = | - | = | = | C | = | = | - | - |
| L | = | C | + | = | + | = | - | C | - | = | - | = |
| N | = | C | = | = | = | = | = | C | = | = | + | = |
| O | = | C | + | = | + | = | = | C | = | = | - | - |
| P | - | C | + | = | + | = | = | C | = | = | = | = |
| Q | - | C | - | - | - | - | = | C | - | - | - | - |
| R | = | C | + | = | + | = | = | C | + | = | + | = |

“C” indicates expression level in WT control either in B or D. “-“, “_+_” and “_=_” correspond to down regulation, up regulation and no changes of expression, respectively as compared to WT control.
