# Supplementary material for: DNA Methylation and Transcriptomic Changes in Response to Different Lights and Stresses in 7B-1 Male-Sterile Tomato
Source: PLoS One. 2015 Apr 7;10(4):e0121864. doi: 10.1371/journal.pone.0121864 (PMC4388563; doi:10.1371/journal.pone.0121864)
Supplement: S1 File — (DOCX) [file pone.0121864.s009.docx]

**Supporting Table A. Global methylation levels in *7B-1* and WT in different lights.**

| **Light condition** | **WT** | ***7B-1*** |
| --- | --- | --- |
| W | 11.4 | 12.4 |
| B | 14.6 | 13.8 |
| R | 14.6 | 19 |
| D | 20 | 21 |

Values are calculated based on the mean of three technical replicates.

**Supporting Table B. Global methylation levels in *7B-1* and WT in response to ABA, mannitol, and fluridone in B and D.**

| **Sample** | **B** | | | | **D** | | | |
| --- | --- | --- | --- | --- | --- | --- | --- | --- |
|  | **MS** | **ABA** | **Mannitol** | **Fluridone** | **MS** | **ABA** | **Mannitol** | **Fluridone** |
| WT | 9.3 | 9.6 | 11.7 | 21.5 | 19.6 | 40 | 36 | 38 |
| *7B-1* | 12.1 | 8.7 | 17 | 6.9 | 19.2 | 7.4 | 14.2 | 27.2 |

Values are calculated based on the mean of three technical replicates.

**Supporting Table C. Expression changes of characterized genes in *7B-1* in B and D in response to ABA and mannitol.**

| **Genes** | ***7B-1*/MS** | **WT/ABA** | ***7B-1*/ABA** | **WT/mannitol** | ***7B-1*/mannitol** |
| --- | --- | --- | --- | --- | --- |
| Lipase/B | -3.2 | 1 | -4.6 | 1.1 | -4 |
| RNA helicase/B | 1 | 1.2 | 5 | 1.4 | 6 |
| PP2C/B | 1 | 1.3 | 4.3 | 1.7 | 6.2 |
| ATPase/B | -3.2 | -2.7 | -6.4 | -3.4 | -7.1 |
| WRKY/B | 1 | 1.2 | 4.5 | 1.6 | 6 |
| 14-3-3/B | -3.8 | -3.2 | -5 | -4.2 | -5.9 |
| Lipase/D | 1.2 | 1.4 | 1.6 | -3.5 | -4.6 |
| RNA helicase/D | -4 | 1.1 | -6.7 | 1.4 | -7.2 |
| PP2C | 1.5 | 1.4 | 1.2 | -6 | -9 |
| ATPase/D | 1.3 | -2.2 | -3.2 | -3.6 | -3 |
| WRKY/D | 1.5 | 1.3 | 2.7 | 1.7 | 2.4 |
| 14-3-3/D | 1.5 | -2.7 | -3 | -3.2 | -3.7 |

Values are calculated based on the mean of three technical replicates.

**Supporting Table D. Expression changes of characterized genes in *7B-1* in response to 5-azaC.**

| **Genes** | ***7B-1*/MS** | ***7B-1*/5-azaC** | **WT/5-azaC** |
| --- | --- | --- | --- |
| SDD1 | 1.1 | 3.7 | 2.8 |
| Zinc finger | -1.2 | -2.8 | 2.3 |
| Ycf4 | 1.1 | 1.2 | 3 |
| NPH3 | -1.6 | 6 | -1.1 |
| ARF8 | 4.5 | -8.3 | -6 |
| ABF4 | 4 | 6.7 | 2 |
| CRY1 | -1.08 | -1.37 | -1.5 |
| CRY2 | -1.2 | 1.8 | 1.8 |
| PHOT1 | -1.08 | -1.1 | -1.1 |
| PHOT2 | -1.2 | -1.8 | -1.4 |
| HY5 | 2.2 | -4.6 | -1.4 |

Values are calculated based on the mean of three technical replicates.

**Supporting Table E. Expression changes of characterized miRNAs and genes in *7B-1* in response to 5-azaC.**

| **Target candidate** | ***7B-1*/5-azaC** |
| --- | --- |
| mir167 | 7.5 |
| mir390 | 5 |
| D7 tasiRNA | 8 |
| D8 tasiRNA | 1.2 |
| ARF8 | -27 |
| ARF2 | -17.7 |
| ARF3 | -12.5 |
| ARF4 | 2.7 |

Values are calculated based on the mean of three technical replicates.

**Supporting Table F. Expression changes of characterized genes in *7B-1.***

| **Target candidates** | ***7B-1*** |
| --- | --- |
| ARF2 | 3.8 |
| ARF3 | 3.2 |
| ARF4 | 2.4 |
| ARF8 | 4.5 |

Values are calculated based on the mean of three technical replicates.
